# Naturally Occurring Osmolyte, Trehalose Induces Functional Conformation in an Intrinsically Disordered Activation Domain of Glucocorticoid Receptor

**DOI:** 10.1371/journal.pone.0019689

**Published:** 2011-05-16

**Authors:** Shagufta H. Khan, John A. Arnott, Raj Kumar

**Affiliations:** Department of Basic Sciences, The Commonwealth Medical College, Scranton, Pennsylvania, United States of America; University of Crete, Greece

## Abstract

Intrinsically disordered (ID) regions are frequently found in the activation domains of many transcription factors including nuclear hormone receptors. It is believed that these ID regions promote molecular recognition by creating large surfaces suitable for interactions with their specific protein binding partners, which is a critical component of gene regulation by transcription factors. It has been hypothesized that conditional folding of these activation domains may be a prerequisite for their efficient interaction with specific coregulatory proteins, and subsequent transcriptional activity leading to the regulation of target gene(s). In this study, we tested whether a naturally occurring osmolyte, trehalose can promote functionally ordered conformation in glucocorticoid receptor's major activation function domain, AF1, which is found to exist as an ID protein, and requires an efficient interaction with coregulatory proteins for optimal activity. Our data show that trehalose induces an ordered conformation in AF1 such that its interaction with steroid receptor coactivator-1 (SRC-1), a critical coregulator of glucocorticoid receptor's activity, is greatly enhanced.

## Introduction

**Figure 1:**
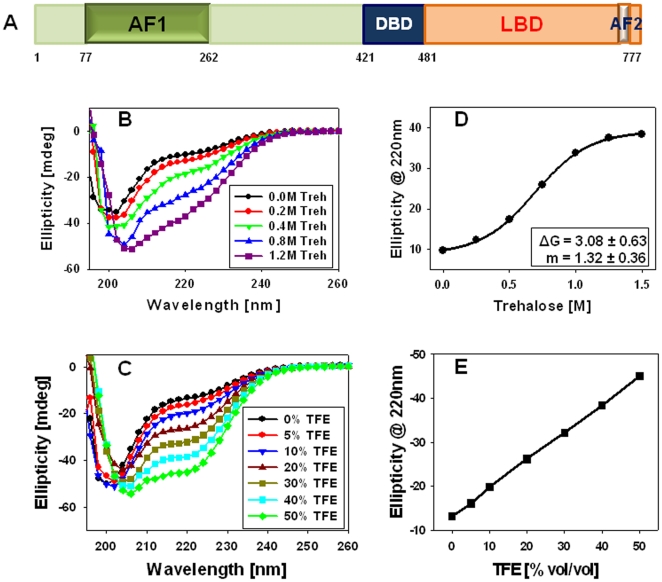
Trehalose induces secondary structural elements in the ID AF1. (A) Diagram of human GR protein showing major functional domains. Far-UV CD spectra of GR AF1 protein in the absence and presence of increasing concentrations of trehalose (B) or TFE (C). (D) Trehalose-induced conformational transition of AF1. The nonlinear least squares best fit of experimental data to two-state model of protein folding/denaturation using linear extrapolation methods gives apparent thermodynamic parameters of trehalose-induced folding: ΔG and m (expressed as kcal/mol; shown in the box). (E) A linear plot showing TFE-induced helical structure in AF1.

Glucocorticoid receptor (GR) mediates biological effects of glucocorticoids at the level of gene regulation [1]. To initiate transcription of target gene(s), GR interacts with its response element DNA, and with various coregulatory proteins [2]. However, specific interaction surfaces of GR with its coregulators are not well understood. Consequently, precisely how transcription is regulated by GR is largely unknown. This is due, in part, to the lack of information about GR's two transactivation function regions, AF1 and AF2 (Figure 1A). AF1 located in the N-terminal domain (NTD) acts in a constitutive manner whereas AF2, located towards C-terminal end of the ligand binding domain (LBD), functions in a ligand-dependent manner [2]. Availability of LBD crystal structure has greatly enhanced our understanding of AF2 function [3]. However, we are only beginning to understand structure and functions of AF1; in spite of the fact that AF1 serves as major transactivation domain for GR. This has hampered our complete understanding of how GR transmits its transcriptional signal from ligand to specific gene(s). The major obstacle in determining AF1 structure has been due to its intrinsically disordered (ID) conformation, frequently found in the activation domains of many transcription factors [4]. It is generally believed that ID regions promote molecular recognition by creating large surfaces suitable for interaction with their specific protein binding partners ([2], [4]). It has been hypothesized that conditional folding of AF1 is a prerequisite for its efficient interaction with specific coactivators, and subsequent transcriptional activity [5]. In this study, we tested whether a naturally occurring organic osmolyte, trehalose can promote functionally ordered conformation in GR AF1. Our data show that trehalose induces an ordered conformation in AF1 such that its interaction with steroid receptor coactivator-1 (SRC-1), a critical coregulator of GR activity, is greatly enhanced.

## Materials and Methods

### Circular dichroism (CD) spectroscopy

Far-UV CD spectra of AF1 protein in the absence and presence of varying concentrations of trehalose were recorded on a Jasco spectropolarimeter using a 0.1 cm quartz cell, with the bandwidth of 1.0 nm and a scan step of 0.2 nm. Each spectrum presented is the result of five spectra averaged, corrected for the contribution of the buffer, and smoothed. Equilibrium folding curves were fitted to linear extrapolation model (LEM) to determine ΔG and m values as described [6].

### Limited proteolytic digestion

Purified AF1 protein in the absence or presence of increasing concentrations of trehalose was partially digested using sequencing grade trypsin, chymotrypsin, or Endo Gluc-C (Sigma–Aldrich) at 4°C for 15 min at a protein∶enzyme mass ratio of 100∶1. Reactions were terminated by boiling for 5 min, and samples were run on SDS–PAGE and stained by Coomassie Blue.

### Electro-mobility shifting assay (EMSA)

Deoxyribonucleotides probes containing consensus glucocorticoid response element (GRE): 5-CTAGGCTGTACAGGATGTTCT-GCCTAG-3 and 5-CTAGGCAGAACATCCTGTACAGCCTAG-3 were synthesized and annealed. To facilitate detection, sense strand was biotin labeled at 3′ end prior to annealing. Binding of GR500 (a fragment of human GR consisting of entire N-terminal and little beyond DNA binding domain) protein to GRE probes was assessed in a buffer containing 20 mM Tris, pH 7.9, 60 mM KCl, 5 mM MgCl_2_ and 1 mM dithiothreitol plus various concentrations of trehalose (0.0–1.2 M). Protein∶DNA complexes were separated on 7% NuPAGE Novex Tris-Acetate gel (Invitrogen), and biotinylated probes were detected using the Pierce Chemiluminescent Nucleic Acid Detection Module (Rockford, IL).

### Immunoprecipitation

HeLa nuclear extracts (NE; 0.5 mg protein), Protein A/G–agarose beads conjugated to SRC-1 antibody were incubated for 4 h at 4°C. AF1 (10 µg) was added and incubated for another 2 h. Beads were washed thoroughly, re-suspended, and boiled to release bound proteins. Released proteins were resolved on SDS–PAGE, and immunoblotted using an AF1 antibody after transferring onto a PVDF membrane as described [5].

### Cell Viability and Reporter gene assays

CV-1 cell viability was determined using WST-1 cell viability assay as per manufacturer's protocol (Roche, Mannheim, Germany). GR AF1 activity was determined using a secreted alkaline phosphatase (SEAP) assay as described [7]. CV-1 cells (lacking endogenous GR) were cotransfected with an expression vector for GR500, a constitutively active form of the human GR [7], a GRE-SEAP promoter∶reporter plasmid, and/or SRC-1 using Lipofectamine plus (Invitrogen) according to the manufacturer's protocol. 24 h later, medium was tested for the presence of SEAP (SEAP Detection Kit; BD Biosciences).

## Results

### Trehalose-induces a compact structure in AF1

Relative effects of increasing concentrations of trehalose on AF1 folding were analyzed using far-UV CD spectroscopy (Figure 1B). As expected, the spectrum of AF1 in buffer shows little secondary structural content, as evident from a minimum around 200 nm, similar to previous results ([8], [9]). Addition of increasing concentrations of trehalose resulted in a concentration-dependent increase in minima around 220 nm, and relative red shift in minima toward 208 nm, a pattern suggesting increased helical content in AF1 protein. Under similar conditions, presence of trifluoroethanol (TFE) also resulted in a concentration-dependent increase in helical content (Figure 1C). These results support the hypothesis that AF1 has a propensity to adopt helical structure and presence of trehalose can promote secondary structure formation in otherwise ID AF1 domain.

### Trehalose-induced conformational transition in AF1 is cooperative in nature

An induced protein folding represents a native-like configuration, if conformational transition is cooperative in nature [10]. Trehalose-induced conformational transition in the AF1 is cooperative as shown by a sigmoidal curve by monitoring the shift in Φ_220_ as a function of trehalose concentration (Figure 1D). On the other hand, similar plots from TFE-induced conformational changes shows a linear trend (Figure 1E), suggesting that trehalose-induced folded conformation of AF1 is unique in nature. The linear least squares best fit of experimental data to two-state model of protein folding/unfolding using LEM gives a free energy change (ΔG) and m value of 3.08±0.63 and 1.32±0.36, respectively. It is evident from the data that both ΔG and m values for trehalose to fold AF1 is consistent with cooperativity of conformational transition [10].

### Presence of trehalose causes AF1 to become resistant to proteolysis

**Figure 2:**
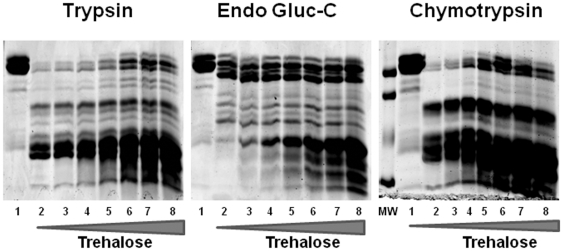
Trehalose-induced folding protects AF1 against partial proteolysis. Products of proteolytic digestion of AF1 resolved on an SDS–PAGE gel after treatment with trypsin (Left Panel), Endo Gluc-C (Middle Panel), and chymotrypsin (Right Panel) in absence (lane 2) or presence of increasing concentrations of trehalose (0.2, 0.4, 0.6, 0.8, 1.0, 1.2 M, lanes 3–8, respectively). MW = Molecular Weight Markers; and lane 1 = undigested AF1.

We carried out limited proteolytic digestions in the presence and absence of trehalose. Patterns of proteolytic products of AF1 after digestion with trypsin are shown in Figure 2 (Left Panel). At 0 and 0.2 M trehalose, protein is nearly completely digested by proteases (lanes 2 and 3), whereas it is partially protected at 0.4 M trehalose and beyond (lanes 4–7). At 1.2 M trehalose concentration, AF1 appears to be maximally protected (Lane 8). 1.2 M is the concentration of trehalose required at which conformational transition reaches saturation, as evident from Figure 1D. Similar results were also obtained with two other proteases, Endo Gluc-C (Figure 2; Middle Panel) and chymotrypsin (Figure 2; Right Panel), suggesting that AF1 has folded into a tertiary structure that moves the residues attacked by these enzymes to positions not easily reached by them. Taken together, these proteolytic digestion data suggest that presence of trehalose induces a compact tertiary structural arrangement in AF1.

### Trehalose-induced conformation facilitates GR's interaction with GRE

**Figure 3:**
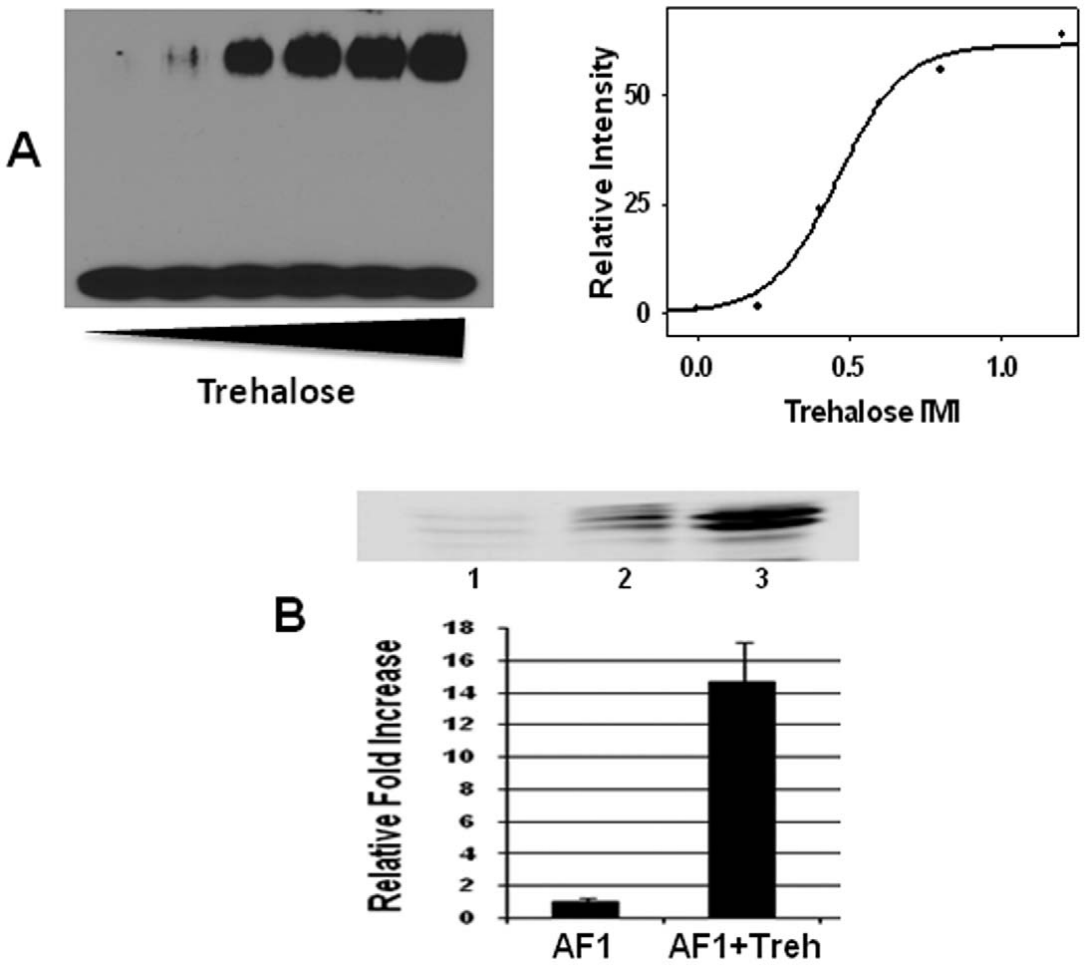
Trehalose-induced conformation facilitates GR's interaction with GRE and SRC-1. (A) Gel mobility shift assay showing binding of GR500 with GRE. Left Panel, a native gel showing bands of GR500:GRE complex; and Right panel, data from densitometric analysis of band of complex plotted against increasing concentrations of trehalose. (B) Immuno-reaction to anti-AF1 antibody after immunoprecipitation (IP) from HeLa nuclear extracts (NE) with SRC-1 antibody (Upper Panel). Lane 1, AF1 + beads (negative control). Lane 2, AF1+NE+beads in absence of trehalose. Lane 3, AF1+NE+beads in presence of trehalose. An average densitometric analyses of band intensity showing relative fold induction (Lower Panel).

In order to determine the functional significance of trehalose-induced folding in GR AF1; we tested whether presence of trehalose affects the binding of GR to its response element, GRE. Using gel mobility shift assay, we determined the binding of GR500 to a consensus GRE sequences. It has been reported that GR500 is capable of binding to GRE and can stimulate AF1-mediated GR activity similar to those observed with full length GR bound to steroid/hormone [7]. Our gel mobility shift data shows that binding of GR500 to GRE is significantly enhanced in the presence of increasing concentrations of trehalose (Figure 3A; Left Panel). Quantification of bands of protein∶DNA complex and plotting them with respect to trehalose concentration gave a sigmoidal curve (Figure 3A; Right Panel) similar to the folding curve of AF1 (Figure 1D). These results suggest that trehalose induced folding may be important for the interaction of GR with its response element DNA.

### Trehalose-induced AF1 conformation facilitates its interaction with a critical coactivator protein

**Figure 4:**
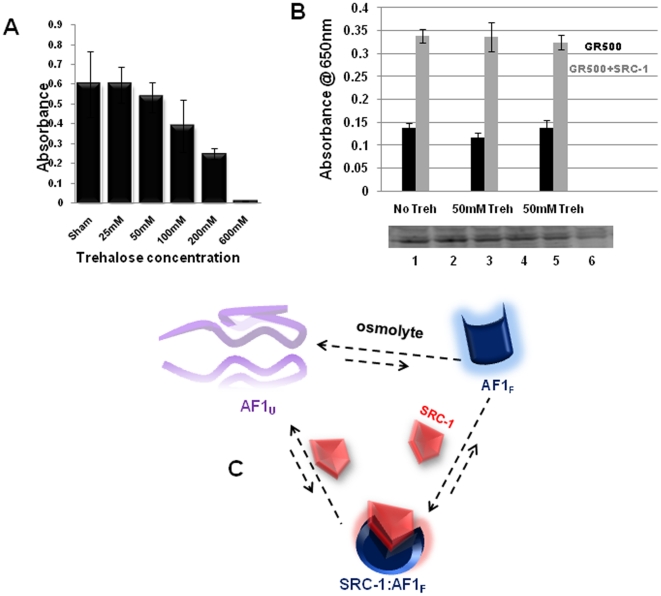
Effect of enhanced interaction of SRC-1 on GR AF1 activity. (A) CV-1 cell viability with or without trehalose treatment (24 h). (B) Upper Panel: AF1-dependent GR-mediated transcription activation of a promoter containing 3×GRE. CV-1 cells constitutively expressing AF1 in a two domain GR fragment containing entire N-terminal and DNA-binding domains (GR500) were cotransfected with DNA of the pGRE_SEAP plasmid alone or plus DNA for SRC-1 in the absence and presence of 50 mM trehalose. Lower Panel: Western blot showing the level of expression of GR500 in each case. In lanes 3 and 4, cells were treated with trehalose 4 h prior to transfections whereas in lanes 5 and 6, 4 h after transfection. Experiments were performed in triplicate. (C) Model for osmolyte-induced folding of GR AF1 domain. AF1U represents the assembly of unfolded conformers of AF1. AF1 could exist only in its unfolded state(s) or in equilibrium with a properly folded state (AF1F). SRC-1 could induce folding directly to AF1U by binding, shifting AF1 directly to the hetero-dimer AF1F·SRC-1. Alternatively, AF1U could be in equilibrium with its native folded state AF1F. Without SRC-1, [AF1F] is relatively small compared to [AF1U]; however, osmolyte could shift equilibrium in favor of AF1F and eventually shift AF1 to the complex, by the law of mass action.

We have previously shown that conditional folding of AF1 is important for specific protein-protein interactions ([5], [11]). Using an immunoprecipitation assay, we evaluated whether conformation induced by trehalose is important for AF1's interaction with its coactivator, SRC-1. In the absence of trehalose, a very small amount of AF1 was found to have precipitated with anti-SRC-1, consistent with our previous data [5]. In the presence of trehalose, a strong interaction between AF1 and SRC-1 proteins occurred (Figure 3B; Upper Panel). Densitometric analysis revealed significant enhancement (∼14 fold) of interaction between AF1 and SRC-1 in the presence of trehalose when compared to samples without trehalose (Figure 3B; Lower Panel). These data indicate that trehalose-induced folding in AF1 facilitates AF1's interaction with SRC-1. We further examined the effects of trehalose-induced functional interaction of SRC-1 and GR AF1 using GR-responsive promoters, in transient transfection-based reporter assays in GR-deficient CV-1 cells. We first established conditions for the incubation of CV-1 cells with trehalose. Cells were incubated for 24 h at various concentrations of trehalose up to 0.5 M. Our cell viability results show that beyond 50 mM concentrations, trehalose is toxic to cells (Figure 4A). Therefore, we selected this concentration of trehalose for cotransfection studies. CV-1 cells were cotransfected with a GRE-SEAP, and GR500 expression vector alone or with added SRC-1. As expected, GR500 alone significantly increased reporter activity (Figure 4B). Input of the plasmid expressing SRC-1 gene enhanced GR500 induction of GRE-SEAP reporter by several fold (Figure 4B). However, incubation of cells with trehalose (50 mM; either 4 h prior or 4 h after transfection) did not significantly alter AF1-mediated promoter∶reporter activity both in the presence and absence of SRC-1 (Figure 4B).

## Discussion

Signals passed by GR from ligand to affect the transcription of specific set of gene(s) depend on GR AF1's interactions with critical coregulatory proteins ([5], [12]). We and others have shown that conditional folding of AF1 region is a prerequisite to facilitate AF1's interaction with specific coregulatory proteins, and subsequent AF1-mediated GR's transcriptional activity ([5], [12], [13], [14]). Because of the ID nature of AF1, we hypothesize that this, in part, is due to the nature of available malleable surfaces in AF1 that arise from differential folding of AF1. Further, factors that can influence AF1 folding should play an important role in its interaction with other coregulatory proteins and consequently in AF1's activity. That raises the question: what are the factors that can bring such folding in AF1 under physiological conditions? Answer to this question, in part, may be explained by the molecular crowding of cells including naturally occurring osmolytes ([15], [16]). Among osmolytes, a disaccharide, trehalose is an interesting molecule. Due to its unique chemical property, this molecule has been the focus of study in several neurodegenerative diseases, associated with the misfolding of disease-specific proteins, including Alzheimer's disease, Huntington's disease, and muscular dystrophy [17]. One of the fascinating aspects of trehalose is its ability to maintain three-dimensional structure of biologic molecules under stress, and thereby preserving their biologic functions [18].

Therefore, in the present study, we determined whether trehalose is capable of inducing a functionally active conformation in GR AF1 such that AF1's interactions with specific coregulatory proteins are facilitated. Although, concentrations of trehalose used here *in vitro* may seem high compared to average intracellular conditions, it has been calculated that osmolyte concentrations in whole tissues often reach 400 mmol/kg of cell water [19], meaning that in certain cellular compartments, concentrations are almost surely much higher. In some species or cells, molar osmolyte levels are reached [20]. Thermodynamic parameters calculated are consistent with the ability of trehalose to fold AF1 such that it displayed significantly increased binding to SRC-1, a critical coregulatory protein for GR action. Predictive algorithms for secondary structure suggest that GR AF1 has the potential for helical structure. It is well known that osmolytes induce protein folding due to solvophobic effects on peptide backbone, and the fact that protein backbone comprises the most numerous functional groups of proteins, osmolyte-induced conformations result in native-like functional species [21]. Effects of trehalose in promoting two important GR functions (interactions with GRE and SRC-1) are testimony to the fact that trehalose-induced structure in AF1 is functionally active.

Our data clearly indicate that trehalose promotes AF1 folding consistent with native-like conformation. On the other hand, TFE is known to promote helical structure in most peptides/proteins, even though it may be non-native helical structure whereas osmolytes increase the driving forces for protein folding ([21], [22], [23], [24]). Due to its solvophobic effect on the backbone, osmolytes force thermodynamically unstable proteins to fold without altering the rules for folding to a native-like conformation ([21], [22]). Conformational transition of GR AF1 observed in TFE can be described in terms of a “random coil” to “α-helix” transition. In contrast to trehalose-induced transition, which is cooperative, TFE-induced conformational change is non-cooperative, typical for helix induction curves described for peptides in TFE/H_2_O mixtures ([23], [24]). Thus, biologically relevant structure is likely to be induced by osmolytes such as trehalose that have been selected in nature for their ability to fold and stabilize proteins.

SRC-1 is reported to be a critical coactivator for GR activity; therefore, enhanced binding of SRC-1 with trehalose-induced folded form of GR AF1 suggests that conditional folding of AF1 is critical for its interaction with specific coactivators. If so, this enhanced binding should enhance GR AF1 transcriptional activity in a promoter∶reporter assay. However, our transient transfection data indicate that in the presence of trehalose (50 mM), both basal as well as SRC-1 mediated GR AF1 activity in CV-1 cells cotransfected with GR500 and SRC-1 mammalian constructs were not significantly altered. One possible explanation for this effect could be the effective concentration of trehalose in cells after incubation with 50 mM. It is important to note that only a small fraction (0.5%) of disaccharides is likely to be absorbed by cells [25]. Further, in mammalian cell culture, intracellular trehalose concentration is dependent on extracellular concentration [26]. There are reports showing that in cells treated with trehalose, intracellular trehalose concentration can be maintained for an extended period of time [27], suggesting that effects of trehalose on the structure and functions of proteins can persist for relatively extended period. None-the-less, our data show that trehalose is capable of inducing functionally active conformation in GR AF1, which is well suited for its interaction with critical coregulatory proteins. Based on our data, we propose a model for possible mechanism of action for GR AF1 (Figure 4C).
